# Supplementary material for: Prescribing errors and associated factors in discharge prescriptions in the emergency department: A prospective cross-sectional study
Source: PLoS One. 2021 Jan 12;16(1):e0245321. doi: 10.1371/journal.pone.0245321 (PMC7802932; doi:10.1371/journal.pone.0245321)
Supplement: S1 Appendix — (DOCX) [file pone.0245321.s001.docx]

**S1 Appendix - Data collection tool**

**NCC MERP Taxonomy of Medication Errors**

- **Error status:**
- **Error.**
- **No error.**
- If **NO ERROR** found the form will not be completed.

**PATIENT INFORMATION**

1. Identification in
   - File Number:
   - Initials:
2. Age or Date of Birth:
3. Gender:

- Female
- Male

1. Weight **[may be omitted unless directly pertinent to the error (e.g., medication overdose in a pediatric patient)]**.
2. Weight in Kg:

**EVENT DATE**

- Weekday.
- Weekend.
- Holiday.

**TIME OF ERROR**

- Morning shift (from 7:30 AM – 3:30 PM).
- Evening shift (from 3:30 PM – 11:30 PM).
- Night shift (from 11:30 PM – 7:30 AM).

**SETTING** (of initial error – the error occurred from which department in ED?)

- Acute Care emergency unit.
- Pediatric emergency.
- Emergency rescue unit.
- Triage emergency.
- Emergency flu clinic.
- OBG emergency.
- University staff emergency.
- Unknown.

**TYPE (Multiple selections can be made if found)**

- **Prescription errors:**
- Dose omission. (if a medication has not been prescribed)
- Wrong Patient.
- Improper Dose.
  - Resulting in over dosage.
  - Resulting in under dosage.
  - Extra Dose.
- Wrong Strength/Concentration.
- Wrong Drug.
- Wrong Dosage Form
- Wrong Route of Administration.
- Wrong time.
- Wrong Frequency.
- Wrong Duration.
- The drug is not indicated (drug does not treat the diagnosis or not indicated for such use)
- **Prescribed Medication Monitoring Error (includes Contraindicated Drugs)**
- Pregnancy-Drug contraindications.
- Drug-Drug Interaction
- Documented Allergy
- Drug-Disease Interaction
- Clinical (e.g., liver function, kidney function, blood glucose, prothrombin, blood pressure, etc.)

**Error Description:**

**……………………………………………………………………………………………..…..………………………………………………………..……..…………………………………………………………..……..………………………………………………………..……..…**

**DESCRIPTION OF EVENT**

- This section is not applicable (wrong patient).
  **( If not applicable – Wrong patient - DO NOT fill this section)**

**This is a free text entry field. The user should provide a narrative description of the event, including how the error was perpetuated and discovered. Other relevant information should be included**, such as:

- Relevant laboratory data or tests, including dates – if applicable –

…..…………………..……………..…………………………………………………………..……..………………………………………………………………………..

- Allergies:
  - Yes.
  - No.
  - If yes, please specify:…………………………………..
- Concomitant therapy with a relation.

…..…………………..……………..…………………………………………………………..……..………………………………………………………………………..

- Indication for use (Diagnosis)

…..…………………..……………..…………………………………………………………..……..………………………………………………………………………..

- Medical intervention(s) following the error
- Accepted.
- Rejected.
- Actions taken and recommendation for prevention

…..…………………..……………..…………………………………………………………..……..………………………………………………………………………..

**DESCRIPTION OF EVENT**

**……..…………………………………………………………..……..………………………………………………………..……..………………………………………………..…………………………………………………………..……..………………………………………**

**PRODUCT INFORMATION**

- This section is not applicable (wrong patient).
  **( If not applicable – Wrong patient - DO NOT fill this section)**

**THERAPEUTIC CLASSIFICATIONS**

- Antibiotic
- Anti-fungal
- Antiviral
- Antacid
- Antihypertensive
- Antiemetic
- Electrolyte replenishes
- Anticoagulant
- Diuretic
- Neuroleptic
- Thyroid replacement therapy
- Antihistamine
- Analgesic, antipyretic, anti-inflammatory (Painkillers)
- Anti-infective and anti-inflammatory, ophthalmic use
- GI agents (Laxatives, Antidiarrheal, Antispasmodics)
- Bronchodilator & Respiratory Agents
- Anti-platelet
- Anticonvulsant
- Antidepressant
- Hormonal replacement therapy
- Anti-diabetic agent
- Immunosuppressive (e.g., Cortisone ...etc.)
- Nasal decongestant (Systematic & Topical)
- Cough relief medications
- Mouth, Throat or dental agents
- Allergy relief medications (Systematic & Topical)
- Other …………………
- Not applicable (wrong patient).

**GENERAL**

- This section is not applicable (wrong patient).
  **( If not applicable – Wrong patient – DO NOT fill this section)**

[Select and complete as many items as possible in this section].

**Name of Drug:**

**Strength:**

**Dose, Frequency & Route:**

**DOSAGE FORM**

- Tablet
- Extended-release tablet
- Capsule
- Extended-release capsule
- Oral Liquid
- Injectable
- Cream-Ointment-Gel-Paste
- Aerosol (spray and metered)
- Other

**PERSONNEL INVOLVED**

Initial Error Made by [Select one item or more if applicable]

- Physician (General practitioner)
- Physician (Specialist)
- Intern
- Resident
- Nurse
- Other …………………
- Unknown (Missing Data)

Error Discovered by [Select one item]

- Pharmacist
- Pharmacy Technician

**CAUSES**

[Indicate the reported causes of the medication error, as stated by the perspective of the reporter of the incident. Select as many causes as are applicable from each section]

- **COMMUNICATION**
- Verbal miscommunication
- Written/electronic miscommunication
- Misinterpretation of the order
- **NAME CONFUSION**
- **HUMAN FACTORS**
- Knowledge Deficit
- Performance Deficit
- Miscalculation of Dosage
- Computer Error
- Undertrained to use the electronic system correctly.
- Incorrect selection from a list by a computer operator
- Inadequate screening for allergies, interactions, etc.
- Transcription Error
- Stress (high volume workload, etc.)
- Fatigue/Lack of Sleep
- **UNKNOWN**

**CONTRIBUTING FACTORS (SYSTEMS RELATED)**

[Select as many items as are applicable from this section].

- Lighting
- Noise Level
- Frequent Interruptions and distractions
- Training
- Staffing
- Lack of availability of health care professional
- Policies and procedures
- Communication systems between health care practitioners
- Pre-printed medication orders
- Other
