# Supplementary material for: Prescribing errors and associated factors in discharge prescriptions in the emergency department: A prospective cross-sectional study
Source: PLoS One. 2021 Jan 12;16(1):e0245321. doi: 10.1371/journal.pone.0245321 (PMC7802932; doi:10.1371/journal.pone.0245321)
Supplement: Attachment — Submitted filename: response to reviewers_PE_28October2020.docx [file pone.0245321.s002.docx]

We want to thank the editor and the reviewers for the time and effort they spent on reviewing our revised manuscript entitled **“Prescriptions Errors Prevalence and Associated Factors in an Emergency Department: A Prospective Cross-Sectional Study"**, their valuable and insightful comments have improved our manuscript substantially.

**Reviewer #4 Comments to Author**

*The study highlights key health issue in the region*

**Comment 1:** *For a start, I suggest the authors to quote and describe previous similar studies (done in the region) in the introduction section. There are two or three studies in Iran, one in Oman etc. There is a more recent systematic review about MEs in Middle East (2019), the one referenced in text is published in 2013. Link: https://link.springer.com/article/10.1007/s00228-019-02689-y*

**Response:** Thank you for suggesting adding to the introduction similar studies and a recent systematic review. Now, we have added these references to the introduction (Page 4, lines 99-101, 107-110).

**References:**

1. Izadpanah F, Nikfar S, Imcheh FB, Amini M, Zargaran M. Assessment of frequency and causes of medication errors in pediatrics and emergency wards of teaching hospitals affiliated to Tehran University of Medical Sciences (24 Hospitals). Journal of Medicine and Life. 2018;11(4):299.

2. Thomas B, Paudyal V, MacLure K, Pallivalapila A, McLay J, El Kassem W, et al. Medication errors in hospitals in the Middle East: a systematic review of prevalence, nature, severity and contributory factors. European journal of clinical pharmacology. 2019;75(9):1269-82.

**Comment 2:** *The current status of results do not provide enough insight into the problem of PEs in Saudi Arabia. One way is to revisit data analysis, which can be improved by investigating the predictors for PEs. For example, class of prescribers, length of stay in ED, number of medications in prescription order.. etc.*

**Response:** Thank you for this comment. We totally agree with the reviewer; however, this information is not available for the researchers and we are limited by the data we have in this study.

**Comment 3:** *Line 103: Ref. 13: There is more recent review about MEs (as mentioned above).*

**Response:** Thank you for pointing this out, now we have added the recent systematic review to the introduction (Page 4, lines 107-110).

**Comment 4:** *Line 196: SPSS should be referenced in text as (IBM Corp., Armonk, N.Y., USA).*

**Response:** Now we have referenced SPSS in the text, thanks (Page 8, line 200).

**Comment 5:** *Table 1: number and % does not correlate with each other. For PE type (wrong dose): 19/82 = 23% but in table is 20.7%!!*

**Response:** Thank you for this comment as it made us revisit the original data and found missing categories. Now the table is updated as well as the % in the abstract (Page 19, Table 1; Page 2, lines 50-51).

**Comment 6:** *Table 2: It might be good to group all oral formulation as one (capsule, tablet etc..)*

**Response:** Thank you for the suggestion; we have made the suggestion to Table 2.

**Comment 7:** *Table 4: Title should be changed to (Healthcare professionals involved in PEs)*

**Response:** Thank you for the suggestion. Now we have made the suggested changes in Table 4.

**Comment 8:** *Appendix: It would be useful to look at the data collection tool used for this study, to be included as appendix.*

**Response:** Thank you for the suggestion. Now we have added the data collection tool (Appendix I) (Page 22).

**Reviewer #5 Comments to Author**

*The authors have mostly addressed the reviewers' comments satisfactorily. However, there a couple of issues remain:*

**Comment 1:** *One of the reviewers raised the issues of the denominator. This is still confusing in the manuscript. From what I can see, there were 504 prescriptions assessed, there were 82 errors in 68 prescriptions. But the number of patients for whom the 504 prescriptions were written is not presented. I suggest this be inserted in the first sentence of the Results e.g. A total of 504 prescriptions for xx patients were assessed.*

**Response:** Thank you for pointing this out. Please note that the total number of patients (Prescriptions) included in the study is 504 out of this number 68 Patients (Prescription) was identified to have errors; therefore, each patient had one prescription. Now we have added this to the results section as well as the abstract (Page 9, lines 204-205).

**Comment 2:** *Results, second sentence: "The total number of errors per patient was 82 (ratio 1:205)" . Firstly, it's impossible there were 82 errors per patient. There was a total of 82 errors. How many patients were there? Furthermore, "1:205" cannot be correct. I think the reviewer's suggestion was 1.205 - i.e. 1.205 errors per patient (not 82 errors per patient) - is this correct?*

**Response:** Thank you for noticing this, we have rephrased the sentence now (Page 10, Line 243)

**Comment 3:** *Table 1 states the total number of PEs is 82, but the addition of the types of errors is 77. If this table is counting PEs, the total should be 82. Please check.*

*- Table 2 - one reviewer raised the issue of the numbers not adding up to the total number of errors. The explanation given by the authors for this discrepancy is satisfactory, but needs clarification within the table by a footnote as readers will ask the same question. Same for Table 3 where the system related factors don't add up to 82.*

**Response:** Now the table is updated as well as the % in the abstract (Page 19, Table 1; Page 2, lines 50-51).

Regarding Table 3, we have added a footnote to clear the confusion (Page 21, Table-3).

**Comment 4:**  *When presenting the results as a prevalence it is ambiguous what the denominator is. I suggest revising this to be e.g. 13.5% of prescription had at least one error, rather than saying a prevalence of 13.5% (as in Discussion first paragraph). This also applies when discussing other studies e.g. Discussion, paragraph 1 - prevalence of 13.4%... is this 13.4 errors per 100 orders or 100 patients, or 13.4% of prescriptions with at least one error?*

**Response:** Thank you for the correction. We totally agree with the reviewer and since we don’t have a denominator to calculate the prevalence, now we have removed the term “Prevalence” and replaced it in the discussion part (Page 10, line 243).

**Comment 5:** *Discussion, line 326-329: The main limitation that should be listed here is the lack of a denominator for the data provided. So for example, the most frequent medications with errors were analgesics and antibiotics, probably because these were the most commonly prescribed drugs. A comparison of error rates would provide a more complete picture of what types of prescriptions were more likely to have errors. However, as the authors have acknowledged that denominators were not available, this should be mentioned as a limitation more generally with the data and not only with the lack of denominators for the age and gender distribution (as it is currently worded).*

**Response:** As per the reviewer’s suggestion, we have added this limitation to the discussion part (Page 14, lines 334-336).

**Comment 6:** *From the Methods, I understand that discharge prescriptions were assessed for errors and not inpatient medication charts. This is based on the Study sample section of the Methods. If so, I think this should be made explicit in the Abstract and even the title.*

**Response:** Thank you for pointing this out, we have now added “*discharge prescriptions”* to the title as well as the abstract.

**Comment 7:** *Some more minor issues:*

*- Table 1 has an asterisk not addressed with a footnote. Please provide full form of abbreviation PE in the table footnote.*

*- Table 3 has an asterisk not addressed with a footnote.*

*-Table 4 needs the number of errors presented in addition to percentages (as in the other tables)*

**Response:** Thank you for pointing these out. Recommended changes have been made.
