# Supplementary material for: Prescribing errors and associated factors in discharge prescriptions in the emergency department: A prospective cross-sectional study
Source: PLoS One. 2021 Jan 12;16(1):e0245321. doi: 10.1371/journal.pone.0245321 (PMC7802932; doi:10.1371/journal.pone.0245321)
Supplement: Attachment — Submitted filename: response to reviewers_PE_06December2020.docx [file pone.0245321.s003.docx]

We want to thank the editor and the reviewers for the time and effort they spent reviewing our revised manuscript entitled **“Prescribing Errors and Associated Factors in Discharge Prescriptions in the Emergency Department: A Prospective Cross-Sectional Study"**, their valuable and insightful comments have improved our manuscript substantially.

**Editor Comments to Author**

*The reviewer points out that the manuscript requires careful proofreading. Besides her comments, revise the whole text. In several sentences, the wording blurs the meaning.*

**Comment 1:** *Page 9, lines 203-9. It seems clear that 68 prescriptions contained one or more errors. As 11 (16%) of them contained more than one error, the total number of identified PEs was 82. However, the wording is not clear, and specifically the expression “total number of errors per patient” is confusing.*

**Response:** Thank you for pointing this out; now we have improved the clarity of this sentence.

**Comment 2:** *According to the study, 36.8% of the identified PEs occurred in pediatric patients. However, it does not mean that “36.8% of prescriptions in the pediatric setting had at least one PEs”, as the authors assert. (Page 11, line 250)*

**Response:** Thank you for this comment. We have removed “at least one”, as we meant the identified PEs.

**Comment 3:** *In the discussion, there are two paragraphs (page 12, lines 276-81; page 13 line, 304 and following) that could be merged and shortened to avoid redundancy. Both of them describe studies reporting that the presence of pharmacists in ED results in a reduction of PEs. Also, a citation is required to support the first sentence of the paragraph on page 12.*

**Response:** Thank you for pointing this out; we have now merged and shortened these paragraphs.

**Comment 4:** *The limitations of the study are explained in the corresponding section. However, this paragraph should be reorganized. A call for further research is frequently included at the end of the discussion section, but not in the middle of the limitations (page 14, line 329-30). So, it is clear that in previous drafts this point was the end of the section and, later, other sentences were added. Also, it is not a felicitous remark to conclude that “Consequently, these data provide very little information” (line 335). Please, rewrite the whole paragraph.*

**Response:** We agree with the editor; we have removed the call for future research from the limitations part and include it at the end of the discussion part.

**Reviewer #5 Comments to Author**

*The authors have addressed most of the reviewer comments. One remains to be addressed:*

**Comment 1:** *Table 2 - one reviewer raised the issue of* the numbers not adding up to the total number of errors*. The explanation given by the authors for this discrepancy is satisfactory, but needs clarification within the table by a footnote as readers will ask the same question.*

**Response:** Thank you for pointing this out. We have included a footnote explaining this point.

**Comment 2:** *One other minor comment is to explain what is meant by Drops in Table 2. e.g. is this eye drops, nasal drops??*

**Response:** Thank you for noticing this, we have changed it into “Eye Drops”.

**Comment 3:** *Some grammar changes needed (but please check rest of manuscript carefully):*

*-Abstract, Methods, first sentence: "in an ambulatory ED", not "at ambulatory ED".*

*-Abstract, Methods, second sentence: "Data were collected for six month using a customized reporting tool."; not "Data collected for six months period...".*

*-Abstract, Methods, Third sentence: "All patients discharged from the ED with a discharge prescription..."; not "All patients who were discharged from ED with a discharged prescription..."*

*-Abstract, Results, first sentence: "504 prescriptions for 504 patients were reviewed and 13.5% (n=68) had at least one error" or "13.5% (n=68) of the 504 prescriptions reviewed (for 504 patients) had at least one error".*

*-Abstract, Results, second sentence: move the % to after the error type e.g. "Main PE encountered were wrong dose (23.2%), wrong frequency (20.7%)....".*

*-Abstract, Conclusion: "PEs in the ED are common...", not "PEs in ED setting is common..."*

*-Introduction, page 4, lines 99-101 (new text): "... revealed that 50.5% of the total MEs occurred in the ED, with 22.6% of these being PEs."*

*-Introduction, page 4, lines 107-109 (new text): the sentence beginning "A recent review..." is not a full sentence, pls revise.*

*-Discussion, first sentence: "This study found that 13.5% of patients' electronic prescriptions in the ED had at least one PE." End the sentence here then compare to the literature in a new sentence, as this is currently a very long sentence.*

*-Discussion, first sentence: "... where PEs were found to occur in 13.4% to 50.5% of prescriptions."*

*-Discussion, first paragraph, last sentence: "...by several studies, but PEs are nonetheless alarmingly common in the ED".*

*-Discussion, last paragraph, line 333-335: "For example, a comparison of error rates for each medication class would...".*

**Response:** Thank you for the comments on the grammatical changes. All changes have been made and highlighted.
